# Evaluation of software for mental health promotion of undergraduate nursing students in the early years of college

**DOI:** 10.1590/1980-220X-REEUSP-2022-0006en

**Published:** 2022-09-19

**Authors:** Caíque Rossi Baldassarini, Jamila Souza Gonçalves, Tania Aparecida Cancian Masella, Jaqueline Lemos de Oliveira, Jacqueline de Souza

**Affiliations:** 1 Universidade de São Paulo, Escola de Enfermagem de Ribeirão Preto, Programa de Enfermagem Psiquiátrica, Ribeirão Preto, SP, Brazil.; 2 Instituto Federal de Educação, Ciência e Tecnologia do Sul de Minas, Passos, MG, Brazil.; 3 Centro Universitário Barão de Mauá, Ribeirão Preto, SP, Brazil.

**Keywords:** Students, Nursing, Mental Health, Health Promotion, Estudiantes, Enfermería, Salud Mental, Promoción de la Salud, Estudantes, Enfermagem, Saúde Mental, Promoção da Saúde

## Abstract

**Objective:**

To evaluate mental health promotion software for students in the early years of undergraduate nursing course.

**Method:**

Descriptive study developed with 41 undergraduates from a private higher education institution in an inland city of the state of São Paulo, approved by the Research Ethics Committee. Data collection was carried out remotely from April to October 2021, using a sociodemographic characterization questionnaire and student assessment of the software. The results were analyzed by descriptive statistics.

**Results:**

Most respondents rated the tool and the clarity of its content as excellent. The modules considered most relevant were those related to solving problems with future implications. The students considered the advice very applicable to everyday life and a good correspondence between problem situations and real life.

**Conclusion:**

This type of intervention is configured as one more option in the list of strategies to promote nursing students’ mental health, although it does not replace face-to-face care.

## RESUMO

**Objetivo:**

Avaliar um software de promoção da saúde mental destinado a estudantes dos anos iniciais da graduação em enfermagem.

**Método:**

Estudo descritivo desenvolvido com 41 graduandos de uma instituição privada de ensino superior do interior do estado de São Paulo, aprovado pelo Comitê de Ética em Pesquisa. A coleta de dados foi realizada de forma remota de abril a outubro de 2021, usando um questionário de caracterização sociodemográfica e avaliação dos estudantes acerca do programa. Os resultados foram analisados por estatística descritiva.

**Resultados:**

A maioria dos participantes classificou a ferramenta e a clareza de seu conteúdo como excelentes. Os módulos considerados mais relevantes foram aqueles relacionados à resolução de problemas com implicações futuras. Os estudantes consideraram os aconselhamentos muito aplicáveis ao cotidiano e uma boa correspondência das situações-problema com a vida real.

**Conclusão:**

Essa modalidade de intervenção configura-se como mais uma opção no rol de estratégias de promoção da saúde mental de graduandos de enfermagem, ainda que não substitua o cuidado face-a-face.

## RESUMEN

**Objetivo:**

Evaluar un software de promoción de la salud mental dirigido a estudiantes de los primeros años de graduación en enfermería.

**Método:**

Estudio descriptivo desarrollado con 41 estudiantes de graduación de una institución de enseñanza superior privada del interior del estado de São Paulo, aprobado por el Comité de Ética en Investigación. La recolección de datos se realizó de forma remota de abril a octubre de 2021, utilizando un cuestionario de caracterización sociodemográfica y evaluación de los estudiantes del programa. Los resultados fueron analizados por estadística descriptiva.

**Resultados:**

La mayoría de los encuestados calificaron la herramienta y la claridad de su contenido como excelentes. Los módulos considerados más relevantes fueron los relacionados con la resolución de problemas con implicaciones futuras. Los alumnos consideraron los consejos muy aplicables a la vida cotidiana y una buena correspondencia entre las situaciones-problema y la vida real.

**Conclusión:**

Este tipo de intervención es una opción más en la lista de estrategias para promover la salud mental de los estudiantes de enfermería, aunque no reemplaza la atención presencial.

## INTRODUCTION

Admission to university is a critical period for the student’s academic development and adjustment, and this experience, in general, occurs concomitantly with the psychological tasks of the transition from adolescence to adulthood – such as the need to develop new responsibilities and autonomy – thus constituting a challenge to be overcome by the student^(1–2)^.

The challenges associated with the recent COVID-19 pandemic are also highlighted, in which isolation measures greatly interfered in several psychosocial aspects, with direct implications for the way of interacting with people and contexts. Regarding students, the necessary adaptations in the learning processes are also emphasized, given the predominance of non-face-to-face teaching in this period. These factors, associated with the fear of the disease, which was also strongly emphasized by the media, are of paramount importance to be considered in terms of the response of undergraduates in what regards mental health, especially among first-year students^(3)^.

As for this public, studies prior to the pandemic period^(4–5)^ already highlighted that the increase in academic demands, especially in the initial years of the course, usually culminates in significant levels of stress and impacts on mental health. Among students in the health area, such as nursing, in addition to the large volume of theoretical content, practical activities, contact with patients, study of illness and death processes, and responsibility for people’s lives and health demand even more from the student’s emotional balance^(4–5)^.

In view of the vast scientific production related to the important impacts of academic and psychosocial factors related to the university experience of nursing students on mental health, the need to develop intervention strategies to promote the mental health of these university students is evident, aiming at better adaptation and resilience in the face of higher education challenges^(5)^. This need has become even more pressing in the context of the pandemic.

Among the multiple possibilities of interventions to promote university students’ mental health, studies show that proposals using online resources have the advantages of a lower cost; ability to reach a large population; accessibility and optimization of intervention time, considering that students have a large volume of academic activities and reduced time available for face-to-face programs^(2,6–8)^. Moreover, the frequent difficulty of access to mental health professionals by many students, due to financial reasons, shortage of professionals in the public system, or even due to the stigma still associated with mental health, shall be considered^(7,9)^.

Another aspect favoring the use of online interventions by university students is the wide access and ease of use of devices such as smartphones, tablets, and computers. Known as “digital natives”, the current generation of undergraduate students uses the internet and new technologies for diverse activities, both academic and professional, as well as personal^(10–11)^. However, it should be noted that the wide use of such digital resources by young people, despite their potential, also has limitations. Excessive time using technologies, for example, can lead to dysfunctional behaviors and health damage^(12)^.

Studies evaluating digital strategies for mental health promotion have gained greater prominence in the last two decades, with the popularization of electronic devices^(11)^. In the last five years, studies evaluating the effectiveness of interventions with online resources to university students and non-clinical populations were carried out in countries such as China^(2)^, South Korea^(13)–14)^, Australia, and New Zealand^(7,15)^, with positive outcomes regarding aspects such as stress, depressive and anxious symptoms, life satisfaction, and well-being. In Brazil, however, scientific production on such remote strategies is still incipient^(16)^.

A scoping review article about mobile apps for self-management in mental health gathered publications on the subject between 2015 and 2020 and demonstrated the need for studies on improving these tools functions, reinforcing the importance of building technologies based on surveys that consider their evaluations^(16)^.

Thus, it is understood that there is a solid body of evidence related to stressors and coping mechanisms of university students, including nursing. However, the development and evaluation of mental health intervention and promotion strategies is still a field to be further explored. Furthermore, given the importance of evaluating the target audience’s satisfaction for the constant improvement of interventions, with implications for adherence and applicability, the present study aimed to evaluate mental health promotion software directed to students in the early years of the nursing undergraduate course.

It is understood that the results of this study will certainly contribute to guide future research on this topic, as well as the development and improvement of care strategies for this public, especially due to the possibility of explaining the potential focuses and themes that are more compatible with emotional manifestations, relational and academic context of this public.

Additionally, considering that current health students will be professionals within a few years, good experience with online mental health promotion, as well as the benefits for the students themselves, can help them consider this type of strategy as relevant also in their professional practice, given the growing number of digital approaches in health^(9,11)^.

## METHOD

### Design of Study and Local

This is a descriptive study developed with students in the early years of the nursing course at a private higher education institution in an inland city of the state of São Paulo, Brazil.

### Population, Eligibility Criteria, Recruitment and Sample

In 2021, the aforementioned institution had 75 students enrolled in the full-time shift and 67 in the night shift in the initial years of undergraduate nursing. Eligibility criteria were: being 18 years old or older and regular attendance of academic activities during the study period. All students were invited to participate in the research, 81% met the eligibility criteria and 41 accepted to participate, making up the final sample. As for the refusals, the alleged reason was lack of time and overload of remote activities. Regarding the sample profile, most participants were female (90%, n = 37), self-declared white (71%, n = 29) and were between 18 and 22 years old (68%, n = 28).

To recruit the participants, the researcher created an infographic with brief information about the purpose of the research, benefits, and instructions for participation. Additionally, the researcher also prepared a video in which the study is clearly and briefly explained, reinforcing the invitation to participate. The video was posted on the online video platform *YouTube* and marked as “unlisted”, that is, only students who received the link were able to access it. The infographic and video were shared by class representatives in the classes’ *Whatsapp* group, and fixed on the Student Portal of the institution by the course coordinator.

### Data Collection

Data collection was carried out remotely from April to October 2021. It was structured in two phases, namely, the use of the software by the students and the respective evaluation through a questionnaire.

### Software Description

The software called “Interactive program for nursing students: reflecting on skills to deal with the challenges of the university context”^(17)^ was developed with CNPq funding (Universal Public Notice No. 28/2018, process 422244/2018-0) and registered at the National Institute of Industrial Property (*INPI*) by the USP Innovation Agency (Process No: BR512020002487-4). The software script underwent content validation by two judges (psychologists and university professors of nursing courses) and was applied to a sample of nursing students from a public university, with promising results in relation to their psychosocial health^(18)^.

The software, available both on the web as in application format for android and iOS, was based on the cognitive-behavioral theory with emphasis on problem solving technique and social skills training^(17–18)^. It consists of six modules: 1) establishing new relationships; 2) integration into a group; 3) coping mechanisms; 4) support request; 5) establishment of priorities in daily activities, and 6) reflection on the academic route and future goals^(17–18)^.

The first four modules, structured in the format of problem situations, present alternative answers about the behavior that the student considers to be most similar to his own in circumstances such as those presented. The software provides advice and information about the consequences of such behavior^(17–18)^.

In the fifth module, there is a list of activities for the students to select their priorities in relation to their daily routine and after the selection a graph illustrating the areas with greater emphasis (academic/labor and leisure, which is divided in physical, social and sedentary) is produced. The system then provides advice for a better balance between them. Finally, in the sixth and last module, based on the reflection on their own academic path, the student is encouraged to describe future goals in the personal, professional, and social spheres^(17–18)^.

## SOFTWARE EVALUATION

### Questionnaire Items

A questionnaire prepared by the authors was used, in electronic format via *Google Forms*, both for surveying minimum personal indicators (age; sex; self-reported color) and for evaluating the tool used.

A previous study^(19)^ developed a taxonomy for the evaluation of Educational software (TaCASE) based on the ISO 9126-1 and 9241-1 standards in which two major criteria, of quality and usability, are proposed. In the present study, we opted for the development of specific evaluative questions considering both the experiences described by studies that worked specifically with the nursing area^(20,21,22)^, as well as in the items proposed by this taxonomy, selecting those most relevant to the scope of the software mentioned.

**Figure 1:**
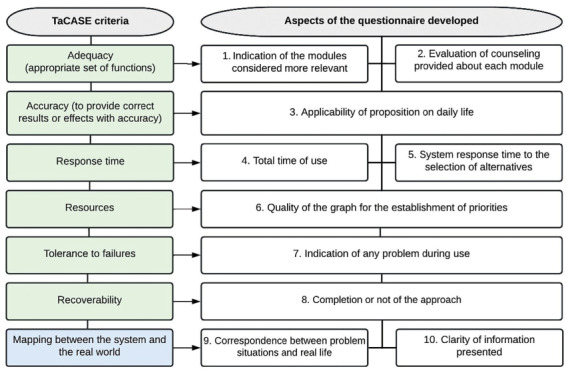
Correspondence of TaCASE with the aspects evaluated by the questionnaire developed by the authors.

Legend: the blue box corresponds to the usability criterion and the green ones correspond to the quality criteria listed by the authors in this study.

Thus, the questionnaire mostly included aspects corresponding to the quality criteria, from which six of the 21 items were listed, and one of the 12 items corresponding to the TaCASE usability criteria, as shown in Figure 1.

In addition to the 10 items shown in Figure 1, two more general questions were added, as proposed in the topic “general impression” of a previous study with nursing undergraduates^20^, with one being called ‘software general assessment’ (item 11) and the other ‘inquiring about possible suggestions for improvements’ (item 12).

### Questionnaire Item Response Methods

**Chart 1:**
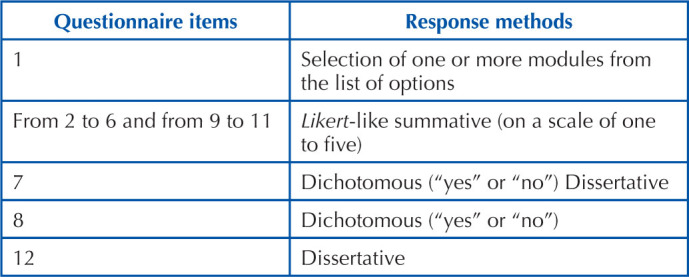
Questionnaire items and corresponding response methods.

As for the response methods proposed in the elaboration of the questionnaire, we opted for the summative method of *Likert* type (on a scale of one to five), as recommended by studies related to this type of assessment^(19–22)^, dichotomous “yes” or “no” format when the question did not imply a value scale, and also a dissertation format with a correspondent field that was optional, when necessary, according to study recommendations on the development of questionnaires in the nursing area^(23)^. The response method corresponding to each item is detailed in Chart 1.

The questionnaire content was analyzed by two judges (nurses, with expertise in mental health) and was used in a pilot study with three undergraduate nursing students participating in the authors’ research group, who positively evaluated the clarity and relevance of the items.

### Data Collection Procedures

The access to the software and the respective evaluation questionnaire was made available through links provided to students in the Student Portal of the educational institution in question, by the coordinator of the nursing course, and also in the classes’ *Whatsapp* groups, by the class representatives. The undergraduates who were interested in participating in the study initially had access to the Free and Informed Consent Form (FICF). Those who agreed to participate indicated their agreement in electronic format, in the *Google Forms*. The software and questionnaire were self-administered.

### Data Analysis

For analysis purposes, we chose to organize the data into three main themes, namely 1) general assessment, 2) interface, and 3) functionality. Theme 1 aggregates the answers referring to the general perception of the participant in relation to the software (items 10 and 11 of the questionnaire); theme 2 corresponds to the user’s interaction with the system (items 4 to 6), and theme 3 concerns the applicability of the proposed themes and advice (items 1 to 3, 7 to 9 and 12).

The participants’ responses were organized in a spreadsheet of the *Microsoft Excel*. This spreadsheet was transferred to the IBM^®^ SPSS version 21.0 software, in which descriptive statistics, dispersion measurements, and graphs were used.

### Ethical Aspects

This study was prepared in accordance with the guidelines and regulatory standards for research involving human beings (Resolution No. 466/2012 of the National Health Council) and was approved by Opinion No. 4.382.426 of November 5, 2020 of the Research Ethics Committee of the Ribeirão Preto Nursing School of Universidade de São Paulo. All participants signed the Free and Informed Consent Form (FICF). Students who pointed out suggestions for improvement or occurrence of problems were coded by numbers, according to the order of participation in the study, to protect their anonymity.

## RESULTS

**Figure 2:**
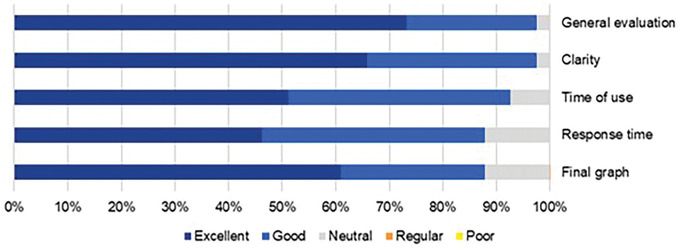
Distribution of participants according to their evaluation in relation to general aspects and the software interface – Ribeirão Preto, SP, Brazil, 2021 (n = 41).

Figure 2 presents the results related to the software general evaluation and the interface.

Of the three items making up the interface, the final graph was the one with the highest percentage of participants who attributed the maximum value of the *Likert* scale on its evaluation (Figure 2).

**Figure 3:**
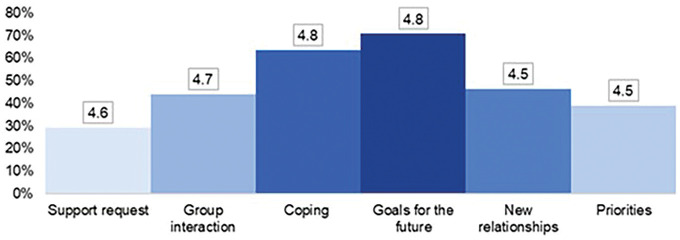
Distribution of participants according to the modules considered most relevant and average of the respective values attributed to counseling in the Likert scale – Ribeirão Preto, SP, Brazil, 2021 (n = 41).

As for functionality, as shown in Figure 3, the modules considered the most relevant and best evaluated in terms of counseling were “goals for the future” and “coping”.

The average of the values assigned, on the *Likert* scale, to the applicability of the propositions in everyday life was 4.4 (sd = 0.62), and the correspondence of problem situations with real life was 4.5 (sd = 0.67).

Regarding the occurrence of problems, two participants indicated the following problems (which did not prevent them from completing all stages of the intervention):

Some parts were a little slow (Participant 8).

I couldn’t click on more leisure activities I like to do (Participant 27).

Four participants indicated that they did not complete the program. However, they did not report the problem that occurred. Five participants suggested improvements:

Maybe at the end, you may put some forms of encouragement to students, words of embracement and support (Participant 4).

I think a dialog box would be great (Participant 22).

If you could make the text smaller, it would be even better. (Participant 31).

The system is great and the design is smart, a quicker response of the system is required (Participant 32).

I think the software could offer more practicality due to the excess of academic activities, with the option of short texts and full texts. In the evaluation of the percentage of activities, it could be possible to insert the averages of hours of each activity for a more precise evaluation. The software has an educational purpose, congratulations (Participant 33).

## DISCUSSION

Regarding the sample sociodemographic profile, there is congruence with the profiles presented in the literature in relation to Brazilian nursing students, namely, a greater number of female students, young people aged between 18 and 24 years, and mostly white^(24–25)^. From a historical point of view, care, characteristic of the nursing profession, is also associated with femininity. Thus, it is common to characterize this work as a female occupation^(24)^. It happens that women often face demands associated with responsibilities with the home and family concomitantly with the university, increasing their stress levels^(5)^. Thus, it is important that the mental health promotion strategies of such academics also consider the specificities related to gender issues.

The literature points out that studies on online strategies for mental health promotion should focus on interventions in which the most unique aspects of the participants are considered, such as personal characteristics and needs, to maintain the adherence of the target audience and maximize the possibility of approach effectiveness^(2,8,11,15–16)^. In this sense, the present study was developed with a very specific group, nursing students in the initial years of graduation, and used software developed especially for this population, considering their particularities and needs. This specificity of the program may, therefore, have contributed to its good general assessment, with the vast majority of students signaling it as “excellent”, in addition to contributing to the indication of satisfactory applicability in the daily life of the propositions presented and correspondence of problem-situations with real life, considering that they were prepared considering specific aspects of adaptation to the beginning of the nursing course.

The assessment of software content clarity as “excellent” or “good” suggests that it presents adequate and objective information, in line with the recommendations of a recent publication on the development of mobile mental health applications, which highlights that interventions requiring less cognitive effort tend to be associated with more effective behavioral changes, optimizing the possibility of the user learning with the platform^(9)^.

The software propositions according to the modules described in the method can be grouped into two large blocks, here called relational (modules 1 and 2) and functional (modules 3, 4, 5 and 6). The first block deals with social skills that can help the student to establish friendship or study relationships in the academic environment. The second block provides guidance on ways to deal with academic situations focused on study, aimed at solving problems, expanding the list of possibilities/strategies for this.

As observed in the results, modules 3 (coping mechanisms) and 6 (definition of goals for the future) were considered the most relevant and also the ones receiving the highest score. It is noted that both modules are part of the functional block, suggesting that such students demand more this type of problem-solving repertoire, despite also benefiting from relational issues.

It is worth mentioning that module 3 dealt with a problem-situation specifically related to a question of academic performance, with a view to future repercussions on their training, a character that is similar to module 6, which also invites the user to visualize him/herself in the future. It is understood that this dynamic of projecting oneself in the future may have, in some way, aroused the interest of the participants, especially because they are first years students who have usually been involved with numerous future projects since the phase of choosing the profession and the course. It should be noted that these characteristics can be considered as an invitation to life projects, which are of paramount importance for strengthening resilience^(26)^.

As far as the interface is concerned, the aspect most widely evaluated as “excellent” was the quality of the user’s tasks distribution graph according to the priorities established on a day-to-day basis. It is noteworthy that this module is the most interactive of the program, in addition to providing a final graph as a product of such interaction. This characteristic is relevant as, in other studies, the interactivity factor was considered especially important for greater adherence to online interventions in health^(7–8)^, and the graphic resources are associated with a lower cognitive effort for learning with the tool^(9)^.

In addition, the availability of a wide list with different leisure possibilities in this module is also configured as a possibility of expanding the student’s behavioral repertoire in this regard. That is, it is understood that with the reading of this list, when selecting those that correspond to their daily lives, as well as with the advice proposed after this selection, the graduating student envisions other leisure alternatives in which he/she can engage. Considering the relevance of physical activities, social interaction and rest to your mental health and well-being^(27,28,29)^, the potential benefits of this repertoire expansion are noted.

Regarding the software response time, previous studies have highlighted the importance of this item in view of its role in motivating the user in relation to the completion of the activity and adherence to the proposal^(8,11)^. In this sense, the present study also corroborates this indication, as observed in the scores achieved by three participants in relation to the slowness of the system when they used the platform. Despite this, the software response time was evaluated as “excellent” or “good” by most participants, which may indicate probable fluctuations in the network accessed by the participant.

Finally, the dissemination and encouragement, on the part of educational institutions, of the availability and use of software or applications previously screened by them, regarding the effectiveness and adequacy of their themes and objectives, were highlighted as a recommendation of paramount importance. That is, the literature points out that university students tend to trust more health applications made available by official and recognized institutions, such as their own universities^(6,11)^, and show greater adherence to software that optimize the time invested, considering the scarcity of free time of this population, often overloaded with academic and/or work activities^(2,30)^. Therefore, it is suggested that new strategies to promote the mental health of undergraduates be added, by a recognized institution, on official platforms, increasing confidence in the intervention and in the safety of its use. This is because, currently, numerous applications have been developed; however, many have a commercial purpose, others provide content that is not scientifically based or even reinforce common sense ideas, especially in relation to mental health^(13,16)^.

As limitations of the present study, it is worth mentioning the use of a self-authored instrument, convenience sampling, and a single educational institution as the study site. It is understood that, although the evaluation questionnaire was submitted to the appreciation of judges with expertise in the area, the use of a validated instrument would increase the reliability of the results obtained. In addition, more statistically accurate sampling, as well as diversification of study sites, would expand the possibilities of generalizing and comparing the results.

The impossibility of recruiting and collecting data in person, due to the COVID-19 pandemic, had direct implications for the sample size. The overload of remote activities and lack of time were aspects highlighted by students who refused to participate. Despite this, the results obtained suggest that the software is a mental health promotion resource also for this public that did not participate in the research, since such a tool overcomes the challenges listed as reasons for refusal, in view of its self-applicable character, its good interface and functionality. Thus, the researchers made links available for use of the software for the entire study population, regardless of whether or not they wished to participate in the evaluation stage.

Finally, it is emphasized that, despite the evident potentialities of the use of online interventions in health, its limitations should not be disregarded. The use of technologies in the daily life of “digital natives”, although essential for the insertion of young people into the dynamism of contemporary society, when in excess, is associated with the loss of relational and emotional management skills^(12)^. Thus, it is understood that the implementation of new digital approaches should be considered within a broader policy of mental health promotion, in balance with face-to-face activities, so that there is no replacement of personal interactions.

## CONCLUSION

The students’ evaluation of the software was very good, especially regarding the general aspects of its use and functionality. It is concluded that the software can be considered as another option among the possibilities of interventions to promote nursing students’ mental health in the early years of college, constituting a promising tool, especially due to its easy access, low cost, and overcoming of barriers such as lack of time for more time-consuming approaches or difficult access to face-to-face strategies.

It is suggested that the elaboration of new proposals to promote nursing students’ mental health should safeguard characteristics such as clarity of content, interactive and responsive interface, use of graphic resources, and availability of the tool by an institution perceived as reliable, to promote adherence and satisfaction from the target audience, with implications for the effectiveness of the intervention. Content focused on issues associated with problem solving is also suggested, and that gender specificities be considered, as most academics are female.

Although no technological tool completely replaces face-to-face care with a trained professional, especially in the case of problems of adaptation and greater emotional distress, the potential of using such resources as an additional strategy is noted, especially among a population of young people considered “digital natives”.
